# Supplementary material for: Quality of life of the cancer patients receiving home-based palliative care in Dhaka city of Bangladesh
Source: PLoS One. 2022 Jul 29;17(7):e0268578. doi: 10.1371/journal.pone.0268578 (PMC9337698; doi:10.1371/journal.pone.0268578)
Supplement: S1 Table — (DOCX) [file pone.0268578.s001.docx]

| **Primary sites of cancer** | **Men**  **(n%)** | **Women**  **(n%)** |
| --- | --- | --- |
|  |  |  |
| Gastrointestinal system | 4 (33.4) | 5 (12.8) |
| Genitourinary system | 5 (41.7) | 7 (17.9) |
| Breast | 0 | 20 (51.3) |
| Endocrine | 0 | 1 (2.6) |
| Eye | 0 | 1 (2.6) |
| Respiratory system | 1 (8.3) | 3 (7.7) |
| Soft tissue | 1 (8.3) | 2 (5.1) |
| Brain | 1 (8.3) | 0 |
